# New Insights on Properties and Spatial Distributions of Skeletal Stem Cells

**DOI:** 10.1155/2019/9026729

**Published:** 2019-06-03

**Authors:** Jun-qi Liu, Qi-wen Li, Zhen Tan

**Affiliations:** ^1^State Key Laboratory of Oral Diseases, National Clinical Research Centre for Oral Diseases, West China Hospital of Stomatology, Sichuan University, Chengdu 610041, China; ^2^Department of Oral Implantology, West China Hospital of Stomatology, Sichuan University, Chengdu 610041, China

## Abstract

Skeletal stem cells (SSCs) are postnatal self-renewing, multipotent, and skeletal lineage-committed progenitors that are capable of giving rise to cartilage, bone, and bone marrow stroma including marrow adipocytes and stromal cells in vitro and in an exogenous environment after transplantation in vivo. Identifying and isolating defined SSCs as well as illuminating their spatiotemporal properties contribute to our understating of skeletal biology and pathology. In this review, we revisit skeletal stem cells identified most recently and systematically discuss their origin and distributions.

## 1. Introduction

Skeletal system, comprised of over 200 individual bones, is essential for general health. Robust skeleton facilitates movement and offers protection for inner organs. Furthermore, mounting evidence showed that the skeleton system is inextricably related with energy metabolism, vascular homeostasis, and immune homeostasis [1–3].

Skeletal homeostasis largely relies on the equilibrium between bone formation mediated by osteoblasts and bone resorption induced by osteoclasts. Perturbation of either of the two processes will cause skeletal disorders. For example, increased bone formation or lack of bone resorption could lead to high bone mass phenotype and reciprocally, excessive osteoclastogenesis or defective osteoblastogenesis can result in diseases like osteopenia, osteoporosis, rheumatoid arthritis, and increased risk of bone fracture [4–7].

Mesenchymal stromal/stem cells (MSCs), main source of osteoblasts, hold great promise for treating skeletal anomalies [8]. Recently, a lot of advancements have made to clarify the mechanism of osteogenic and chondrogenic differentiation of MSCs [9–13]. Over the past few years, many scholars including the concept inventor have been insisting that the term “MSC” should be abandoned or revised due to heterogeneity and overestimated stemness. Under such circumstances, the concept “skeletal stem cells” emerged [14–19].

Mesenchymal stromal/stem cells and skeletal stem cells are two confounding terms for most researchers. Mesenchymal stem cells are referred in most cases and according to the International Society for Cellular Therapy, MSCs should at least meet three minimal criteria: Firstly, they can adhere on plastic when cultured in standard conditions. Secondly, several surface molecules (CD73, CD90, and CD105) should be expressed by MSCs while some other markers should be excluded (CD34, CD45, CD14 or CD11b, CD79a or CD19, and HLA-DR). Thirdly, MSCs must possess trilineage differentiation capacity to osteoblasts, adipocytes, and chondroblasts in vitro [20]. These criteria help researchers identify and isolate stem cells easily. Nevertheless, such definitions are based on in vitro properties and can lead to misjudgment sometimes as in vitro experiments cannot represent in vivo characteristics. For instance, myxovirus resistance-1- (Mx1-) positive population of bone marrow mesenchymal stem cells are tripotent ex vivo (osteoblasts, adipocytes, and chondrocytes) but are defective in chondrogenic and adipocytic lineage differentiation in vivo [21]. By contrast, the definition of skeletal stem cells is more stringent. They are defined as a group of self-renewable cells that are restricted within the skeleton and multipotent to give rise to skeleton-related progenies including osteoblasts, chondrocytes, and adipocytes both in vitro and in an exogenous environment after transplantation in vivo.

Here, a detailed comparison of MSCs and SSCs is provided (Table 1). Firstly, MSCs consist of stem cells of both skeletal lineages and nonskeletal lineages, which means MSCs are distributed ubiquitously [22], while SSCs are inherently restricted to and contribute to skeletal-related tissue including bone, cartilage, bone marrow stroma, and adipose tissue [15, 17]. Secondly, the minimal criteria defining MSCs inevitably lead to cell heterogeneity and variability. Their biological behavior such as colony-forming unit and multipotent differentiation ability varies with donors [23]. In comparison, SSCs are more defined and expected to exhibit more stable properties, largely owing to the discovery of exact cell surface markers as well as a comprehensive in vivo lineage tracing study. Further, SSCs possess multilineage differentiation (osteoblasts, chondrocytes, and adipocytes) capacity both in vitro and in an exogenous environment after transplantation in vivo. Transplantation of SSCs into nonskeletal tissue (e.g., kidney capsule) leads to ectopic bone organoid formation, including bone marrow. Furthermore, serial transplantation of isolated SSCs from the primary donor results in de novo formation of heterotopic ossicles. In comparison, MSCs barely exhibit aforementioned potential [17, 18, 24–26].

In the last decade, the isolation of MSCs was based on their plastic-adherent ability and expression of limited surface markers [20, 27, 28]. Emergence and advancement of research protocols, for instance, combined with the use of fluorescent reporter mouse, lineage tracing, and fluorescence-activated cell sorting (FACS), makes isolation and functional assessment of a precise SSC accessible [29]. Recently, a cohort of candidate markers were identified to label different SSC populations. Most of these populations are self-renewable, clonogenic, and multipotent. In addition, these cells are instrumental in bone injury healing, which is in accordance with the description that a true SSC is capable of responding to injury [30]. At the same time, SSCs in different developmental stages and locations often exhibit distinctive properties. For example, most perivascular SSCs play a role in maintaining hematopoiesis and cranial suture SSCs contribute exclusively to intramembranous ossification. Properties of SSCs change with age too. Together, in this review, we systematically discuss about the recent discovery of SSCs, with specific focus on their origin, stemness, and spatial-temporal variation. Moreover, similarities and differences among these cells are also indicated.

## 2. Growth Plate

The growth plate (or epiphyseal plate) is a type of hyaline cartilage that exists between the epiphysis and metaphysis of a long bone. The growth plate plays a critical role in bone elongation through endochondral ossification [31]. Several growth factors including Indian hedgehog (Ihh), parathyroid hormone-related protein (PTHrP), fibroblast growth factors (FGF), bone morphogenetic proteins (BMP), and vascular endothelial growth factor (VEGF) regulate this endochondral bone formation process [31–35]. Depending on different stages of chondrocytes, the growth plate is divided into a resting zone, a proliferation zone, a prehypertrophic zone, and a hypertrophic zone [32]. A resting zone is considered as an enrichment area of stem-like cells especially chondroprogenitors and sustains the development of the other zones and longitudinal bone growth [36]. A very recent research revealed that a stem cell niche exists in the growth plate of mice, providing new insights into treating children growth disorders [37]. These features make the growth plate an ideal place to find skeletal stem cells.

### 2.1. CD45^−^Ter-119^−^Tie2^−^AlphaV^+^Thy^−^6C3^−^CD105^−^CD200^+^ Cells

Chan et al. isolated cells from femoral growth plates of mice through enzymatic and mechanical dissociation. FACS showed that a large group of cells were CD45^−^Ter-119^−^119^−^Tie2^−^AlphaV^+^ (hereafter termed as [AlphaV^+^]). Subsequent microarray analysis of [AlphaV^+^] further divided this population into eight subpopulations, based on different expressions of CD105, Thy, 6C3, and CD200.

CD45 and Ter-119 are universally expressed in hematopoietic cells. Tie2 is an angiopoietin receptor mostly expressed by endothelial cells and hematopoietic cells. Therefore, CD45, Ter-119, and Tie2 are markers to exclude hematopoietic lineages from bone marrow. AlphaV, as a member of the integrin family, is recently identified as a receptor for irisin, a kind of myokines that promote bone remodeling [38–40]. Thy is a heavily N-glycosylated glycophosphatidylinositol which is expressed on MSCs, fibroblasts, microvascular endothelial cells, neurons, hematopoietic stem cells (HSCs), and mouse T cells [41–43]. CD105 (also known as endoglin) is a type I membrane glycoprotein and a part of the TGF-*β* receptor complex. CD105 can act as a marker of bone marrow colony-forming unit-fibroblasts (CFU-Fs) [21]. The type I membrane glycoprotein CD200 is predominantly expressed on some thymocytes, lymphocytes, neurons, and endothelial and follicular dendritic cells.

Experiment showed that both the [CD45^−^Ter-119^−^Tie2^−^AlphaV^+^Thy^−^6C3^−^CD105^−^CD200^+^] (hereafter short termed as [AlphaV^+^Thy^−^6C3^−^CD105^−^CD200^+^]) subpopulation and single cell sorted from it could generate the other seven subpopulations in a linear fashion both in vitro and in an exogenous environment after transplantation in vivo, indicating that [AlphaV^+^Thy^−^6C3^−^CD105^−^CD200^+^] cells lie at the apex of the skeletogenic differentiation hierarchy [25]. In addition, the [AlphaV^+^Thy^−^6C3^−^CD105^−^CD200^+^] population possesses the ability of self-renewal and multipotency (bone, cartilage, and stroma). Please note that single cell sorted from the [AlphaV^+^Thy^−^6C3^−^CD105^−^CD200^+^] subgroup requires the help of a “supportive niche” to give rise to chondrocytes and osteocytes upon kidney capsule transplantation. In this experiment, 5000 unsorted cells from the long bones were used to provide the “supportive niche.” Without them, the individual [AlphaV^+^Thy^−^6C3^−^CD105^−^CD200^+^] cell cannot survive beneath the renal capsule. Compared with uninjured sites, callus of an injured site had more SSCs and these cells were more osteogenic, revealing a pivotal role of mSSCs in fracture healing. Taken together, researchers conclude that the [AlphaV^+^Thy^−^6C3^−^CD105^−^CD200^+^] cell represents a kind of mouse skeletal stem cell (mSSC) population and that the seven other subpopulations of [AlphaV^+^] are descendants of mSSC [44].

Some factors were identified that could influence the activity and differentiation of the [AlphaV^+^Thy^−^6C3^−^CD105^−^CD200^+^] mSSCs and their progenies. Firstly, Gene Expression Commons analysis of microarray data and single-cell RNA sequencing both indicated that autocrine signaling and/or paracrine signaling are present in this mSSCs and descendants. Secondly, the proliferation of the [AlphaV^+^Thy^−^6C3^−^CD105^−^CD200^+^] mSSCs could be induced by recombinant BMP and inhibited by the BMP2 antagonist in culture. Interestingly, some progenies of the mSSCs expressed antagonists of the BMP2 signaling pathway, such as Gremlin-2 and Noggin, suggesting that downstream skeletal progenitors can regulate mSSC activity.

Fate commitment of these skeletal stem/progenitor cells can be shifted between the bone and cartilage. On the one hand, prochondrogenic progenitors (PCPs or [CD45^−^Ter-119^−^Tie2^−^AlphaV^+^Thy^+^6C3^−^CD105^+^CD200^+^] cells), the skeletal progenitors that are directed primarily toward cartilage formation, can differentiate into a bone when cotransplanted with the bone, cartilage, and stromal progenitors (BCSPs), a progeny of the [AlphaV^+^Thy^−^6C3^−^CD105^−^CD200^+^] mSSCs. On the other hand, VEGF blockade can promote chondrogenesis of SSCs, probably at the expense of osteogenesis. BMP2 can induce de novo formation of [AlphaV^+^Thy^−^6C3^−^CD105^−^CD200^+^] cells in some extraskeletal locations. Considering the aforementioned results, it is understandable that codelivery of the BMP2 and VEGF inhibitor can induce de novo formation of cartilage in adipose tissue.

### 2.2. PDPN^+^CD146^−^CD73^+^CD164^+^ Cells

After identifying a kind of SSCs in the mouse (CD45^−^Ter-119^−^Tie2^−^AlphaV^+^Thy^−^6C3^−^CD105^−^CD200^+^ cells), Chan et al. found that PDPN^+^CD146^−^CD73^+^CD164^+^ cells represent a type of human skeletal stem cell, which can be obtained from fetal and adult bones, BMP2-treated human adipose stroma, and iPSCs [24].

Podoplanin (PDPN) is a conserved mucin-type protein found among species. PDPN can act as a diagnostic marker in certain types of cancer [45]. CD146 (also known as MCAM) is a cell adhesion molecule that closely related with melanoma. A previous study revealed that CD146 can mark a type of self-renewing osteoprogenitors in human bone marrow and the CD146^+^ osteoprogenitors can establish a hematopoietic microenvironment [46]. CD73 is a glycosylphosphatidylinositol-linked cell surface protein and is considered as a potential target of several cancers [47]. CD164 is a mucin-like receptor mainly expressed by CD34^+^ hematopoietic progenitor cells and can suppress hematopoietic cell proliferation [48].

In this experiment, seven distinct cell populations were isolated in the human fetal growth plate based on their different surface expressions of PDPN, CD146, CD73, CD164, and THY1 by FACS. These cells were neither endothelial nor hematopoietic. Among them, PDPN^+^CD146^−^CD73^+^CD164^+^ cells are at the apex of the skeletal lineage hierarchy, with the ability of self-renewal and multipotency (cartilage, bone, and stroma but not fat) in vitro and in vivo. It is noteworthy that PDPN^+^CD146^−^CD73^+^CD164^+^ cells managed to form ectopic ossicles with marrow cavity after serial renal capsule transplantation. Additionally, PDPN^+^CD146^−^CD73^+^CD164^+^ cells can respond to skeletal injury through expansion of cell numbers and cell size. Based on the results mentioned above, PDPN^+^CD146^−^CD73^+^CD164^+^ cells meet the rigorous standards of SSCs [44].

Similar with the mSSCs identified previously, BMP2 can cause de novo bone formation in human adipose stroma (HAS) and the newly formed ossicles housed PDPN^+^CD146^−^CD73^+^CD164^+^ hSSCs and downstream PDPN^−^CD146^+^ human osteoprogenitors (hOPs). Codelivery of the VEGF inhibitor and BMP2 can promote chondrogenesis at the expense of bone formation. Despite similarities mentioned above, differences of the gene expression profile during bone development including WNT, BMP, hedgehog, FGF, and Notch signaling pathways were identified between mSSCs and hSSCs. Some of these genes were exclusively expressed by hSSCs or mSSCs, for example, *SOST*, *CXXC4*, and *DNAJB6* were absent in mSSCs. At the same time, genes like *RUNX2* and *SOX9* were both expressed by mSSCs and hSSCs but showed different activity. The analysis about gene expression partially explains the divergencies on the formation of the ectopic bone and CFUs.

It is noteworthy that there exists a crosstalk between hSSCs and human hematopoietic stem cells (hHSCs). The two groups of cells support each other mainly through cytokines. On the one hand, hSSCs and its subpopulations expressed varieties of hematopoiesis-supportive cytokines such as ANGPT1, CSF1, SDF, IL27, IL7, and SCF, whose matching cognate receptors are expressed on hHSCs and progenies. On the other hand, hHSCs secrete a variety of factors to support the hSSC lineage, such as BMP2, BMP8A, DHH, FGF3, WNT1, and WNT8.

### 2.3. PTHrP-Positive Resting Chondrocytes

As it is widely accepted that stem cells are quiescent before they are needed and the resting zone of the growth plate is abundant in stem cell-like cells especially chondroprogenitors, it seems reasonable to find skeletal stem cells in the resting zone of the growth plate, where PTHrP plays a critical role in delaying hypertrophy of chondrocytes through interactions with Ihh [34, 49]. Based on this assumption, PTHrP^+^ chondrocytes from the resting zone of the postnatal growth plate were identified as skeletal stem cells [50].

PTHrP^+^ cells were distributed in the perichondrial region during a fetal stage. At postnatal day (P) 3, PTHrP^+^ cells appeared at the resting zone. During P6 to P9, they proliferated markedly. The number of PTHrP^+^ chondrocytes peaked at P15 and formed columnar chondrocytes longitudinally that were not restricted in the resting zone. They could gradually extend to primary spongiosa and bone marrow. Lineage tracing showed that besides giving rise to hypertrophic chondrocytes, a fraction of the PTHrP^+^ resting chondrocytes can differentiate into col1a1 (2.3 kb)-GFP^+^ osteoblasts and Cxcl12-GFP^+^ stromal cells in vivo. In contrast, PTHrP^+^chondrocytes ineffectively give rise to adipocytes either in lineage tracing or subcutaneous transplantation but can be induced to adipocytes under adipogenic differentiation conditions in vitro. Under pathological conditions such as growth plate injury, PTHrP^+^ resting chondrocytes lose their physiological fate and directly differentiate into osteoblasts instead.

In addition to multipotency, PTHrP^+^ resting chondrocytes are self-renewing and clonogenic. Interestingly, PTHrP^+^ resting chondrocytes developing before (P9) or after (P12) secondary ossification center formation possess distinct self-renewability. P9 PTHrP^+^ cells failed to survive the third passage while a fraction of P12 PTHrP^+^ cells can survive even after nine passages. Taken together, PTHrP^+^ cells are heterogeneous populations consist of transient, short-term, and long-term skeletal stem cells.

Of note, flow cytometry analysis of PTHrP^+^ resting chondrocytes demonstrates a portion of overlap with the mouse skeletal stem and progenitor cells identified previously by Chan and colleagues but not Gremlin1^+^ cells [25], further proving that PTHrP^+^ resting chondrocytes represent a type of skeletal stem cells from immunophenotypical perspective. Collectively, these observations suggest that PTHrP^+^ resting chondrocytes are a unique type of SSCs.

Probably due to the function of PTHrP and hedgehog (Hh) signaling on delaying hypertrophy of chondrocytes, PTHrP^+^ resting chondrocytes are critical in maintaining the integrity of the growth plate. Partial loss of PTHrP^+^ resting cells is enough to induce premature hypertrophic differentiation of chondrocytes in the proliferating zone. Differentiation of PTHrP^+^ resting cells toward columnar chondrocytes can be repressed regardless of using an agonist or an antagonist of Hh signaling.

### 2.4. Gli1-Expressing Cells

Glioma-associated oncogene 1 (Gli1) is a transcription factor and an effector of the Hh pathways. Gli1 is closely related to osteoblast differentiation and marks MSCs in several organs of adult mice, like craniofacial bones and incisors [51–53]. For instance, a very recent experiment revealed that Gli1 play a key part in mediating Numb-deficient osteoblasts and bone resorption through Hh pathways [54].

Shi et al. discovered that a group of Gli1^+^ cells termed “metaphyseal mesenchymal progenitors” (MMPs) was pivotal for cancellous bone formation. MMPs are located in chondroosseous junction immediately under the growth plate in young postnatal mice. Subsequent genetic lineage tracing experiments unveiled several unique features of MMPs [55].

Firstly, a large number of MMPs were enriched in mRNA associated with some MSC markers, including CD146/Mcam, CD44, CD106/Vcam1, Pdgfra, Pdgfrb, *α*Sma, and Lepr. Secondly, MMPs were at least tripotent to generate osteoblasts, bone marrow stromal cells (BMSCs), and bone marrow adipocytes in vivo. Of note, the experiment data showed that 20% and <10% of Gli1^+^ cells were positive for Osx and Col1, respectively, at 1 month of age and after one-month chasing, the proportion increased to 50% and 80%. Ablation of MMPs reduced the bone mass because of defective bone formation rather than bone resorption, which is evidenced by decreased serum propeptide of type I procollagen (P1NP) and a normal level of C-telopeptide (CTX-I) in Gli1-CreER^T2^;Ai9;Rosa-DTA mice.

It is noteworthy that MMP-derived osteoblasts supported cancellous bone formation mainly at a very young age (juvenile mice, till 4 months of age), while the MMP-derived BMSCs about half of which expressed Lepr (49.1 ± 9.5%, 6 months of age) may took the responsibility for long-term skeletogenesis in adult mice by generating osteoblasts, adipocytes, and bone marrow stroma. As for fracture healing, MMPs can contribute to bone regeneration by promoting bone (~50% osteocalcin^+^ cells) and cartilage (~63% aggrecan^+^ cells) formation. Overall, MMPs can be regarded as a type of SSCs or at least a source of SSCs if not.

Previous studies have revealed the role of Ihh signaling on osteoblast differentiation, and Gli1 is an important transcription factor of Ihh-Smo signaling pathways [51]. Expectedly, blockade of Hh signaling in MMPs caused reduced bone mass and trabecular bone number in juvenile mice without affecting bone resorption. Smo deletion decreased the proliferation of MMPs and impaired their osteogenic differentiation. In addition, conditional knockout of *β*-catenin in MMPs leads to decreased cancellous bone mass and increased marrow adiposity, corresponding with the previous observations on *Osx-Cre;β-catenin^fl/fl^* mice [56]. This result indicates the determinant role of *β*-catenin in the fate commitment of MMPs.

### 2.5. Gremlin 1-Expressing Cells

Gremlin1 (Grem1), as a BMP antagonist and a VEGFR2 agonist, has been recognized that it functions in embryonic and postnatal skeletogenesis [57–59]. Worthley et al. demonstrated that Grem1 may mark a small group of “skeletal stem cells” immediately adjacent to the growth plate. The number of Grem1^+^ cells was rare, only comprised 0.0025% of the live, mononucleated bone marrow cells after collagenase digestion [60]. Distinct from perivascular MSCs like Nestin^+^ cells and LepR^+^ cells which contribute to skeletogenesis mainly in later adulthood, Grem1^+^ cells can function in both development and adult stage, especially in early life [61, 62].

Grem1^+^ cells are clonogenic in vitro and in vivo, and this ability is stronger than Nestin^+^ MSCs. Grem1^+^ cells were tripotent to produce bone, cartilage, and reticular marrow stromal cells, but not fat, in development and adulthood of mice (~64% of the bone and 50% of the chondrocytes of the metaphyseal and epiphyseal bone, at the age of 4 weeks). Thus, the Grem1^+^ cells are termed as osteochondroreticular (OCR) stem cells. Gene expression profile showed that several pathways relating to osteochondral differentiation rather than adipocytic differentiation were elevated in the Grem1^+^ cells. Grem1^+^ cells are highly active in BMP signaling, ECM-receptor interaction, PI3K-AKT signaling, and focal adhesion pathways, which correlates with osteochondral differentiation potential of Grem1^+^ cells. Moreover, Grem1^+^ cells and descendants highly expressed adipogenesis inhibitors. Grem1^+^ cells were critical for bone formation. *Grem1* null mice were osteopenic [58], and an incomplete ablation of Grem1^+^ cells using Grem1-creER^T^;R26-LSL-DTA leads to less total bone volume and trabecular bone fraction of mice. Moreover, Grem1^+^ cells could function in fracture repair by generating osteoblasts and chondrocytes in vivo.

## 3. Perivascular

Mesenchymal cells in hematopoietic niche often provide regulatory cues for HSC development and homeostasis. At the same time, many important discoveries of SSCs are based on vasculature, indicating a function of the vascular microenvironment for SSCs [26, 63–65]. The association between MSCs/SSCs and hematopoiesis is the focus of the study [66]. A crowd of perivascular MSC/SSC markers have been identified, such as Nestin, LepR, Prx1, Mx1, PDGFR, CD51, and CD146 [21, 46, 61, 62, 67, 68]. Nestin-GFP cells, for example, found perivascular in the bone marrow, were capable of trilineage differentiation (osteoblasts, chondrocytes, and adipocytes) and possess SSC-related activities. In addition, Nestin-GFP cells expressed high levels of HSC maintenance genes like *Cxcl12*, *angiopoietin-1* (*Angpt1*), and *interleukin-7*^61^. In this chapter, we will describe three groups of perivascular SSCs in detail. Among them, LepR^+^ cells and CD45^−^CD31^−^Sca1^+^CD24^+^ cells play a regulatory role in hematopoiesis, while the association between Hox^+^ cells and hematopoiesis remains unclear.

### 3.1. CD45^−^CD31^−^Sca1^+^CD24^+^ Cells

Flow cytometric sorting of CD45 and CD31 excludes the hematopoietic and endothelial lineages in bone marrow [69]. Sca1 (stem cell antigen-1), as a mouse glycosyl phosphatidylinositol-anchored cell surface protein, has been commonly used as a marker for HSCs. More importantly, Sca1 is also used in isolating stem/progenitor cells from the skeletal system [70]. CD24 is a mucin-type sialoglycoprotein that is expressed mainly by immature hematopoietic cells [71].

CD45^−^CD31^−^Sca1^+^CD24^+^ cells are mostly located in the perivascular niche and more abundant in the metaphyseal area than diaphyseal area [72]. CD45^−^CD31^−^Sca1^+^CD24^+^ cells possess the following skeletal stem cell-like characteristics. Firstly, it has marked colony-forming unit ability. Secondly, CD45^−^CD31^−^Sca1^+^CD24^+^ cell population had an excellent multipotent capacity to give rise to osteochondrogenic progenitor cells (OPCs: CD45^−^CD31^−^Sca1^−^PDGF*α*^+^) and two subsets of adipogenic populations: fate-committed adipogenic progenitor cells (APCs: CD45^−^CD31^−^Sca1^+^CD24^−^) and a more mature preadipocyte (preAd: CD45^−^CD31^−^Sca1^−^Zfp423^+^). Thirdly, CD45^−^CD31^−^Sca1^+^CD24^+^ cells were able to contribute to bone healing when these cells were transplanted into the defect through generating some osteogenic and chondrogenic structures as with OPCs. The two aforementioned adipocytic populations can delay the healing process, which is at least partially attributed to DPP4 (dipeptidyl peptidase-4), a protease acts commonly as a target of treating diabetes clinically and can be released by CD45^−^CD31^−^Sca1^+^CD24^+^ cells and APCs after adipogenic differentiation. DPP4 inhibitors can promote osteogenic differentiation of CD45^−^CD31^−^Sca1^+^CD24^+^ cells and OPCs, reversing the inhibitory effect of the APCs and preAd on bone healing. Moreover, the adipocytic lineage of CD45^−^CD31^−^Sca1^+^CD24^+^ cells can be influenced by age and diet, with increased accumulation of APCs instead of OPCs

CD45^−^CD31^−^Sca1^+^CD24^+^ cells and adipogenic progenies have distinct effect on hematopoiesis. On the one hand, CD45^−^CD31^−^Sca1^+^CD24^+^ population itself can promote the hematopoietic regeneration in irradiated mice with increased hematopoietic progenitor cells. On the other hand, the transplantation of APCs or preAds could impair hematopoietic reconstitution, which was consistent with the previous view that bone marrow adipocytes act negatively on hematopoietic homeostasis [73].

### 3.2. Leptin Receptor-Expressing Cells

Leptin is a fat-derived hormone that plays a crucial part in regulating appetite and energy expenditure [74]. Moreover, leptin is involved in osteogenesis via central and peripheral pathways [75, 76]. The leptin receptor is a class I cytokine receptor that gradually appears postnatally, and deficiency of it can lead to obesity [77].

Nowadays, leptin receptor (LepR) is widely used to mark SSCs of adult mice as leptin receptor-expressing (LepR^+^) cells occur almost specifically in adult mice [62]. LepR^+^ cells reside around sinusoids and arterioles and significantly overlap with other MSC markers including PDGF*α*, CD51, PDGF*β*, CD105, *Prx1*, and *Nestin*-GFP^low^ but rarely express *Nestin*-GFP^high^. LepR^+^ cells only comprise 0.3% of bone marrow cells but are highly clonogenic, consisting of most bone marrow CFU-Fs (94% ± 4%). LepR^+^ cells are tripotent to give rise to the bone, cartilage, and fat in vitro and upon subcutaneous injection and are a major source of the bone and adipocytes from 2 months of age. The capacity of LepR^+^ cells to generate bone and adipocytes increases with age.

LepR^+^ cells are quiescent physiologically but can be activated upon irradiation or bone fracture. Irradiation activates LepR^+^ cells to give rise to osteoblasts and adipocytes, and LepR^+^ cells are considered as the main source of bone marrow adipocytes of adult mice [62]. LepR^+^ cells can contribute to bone and cartilage healing while chondrogenesis is hardly seen under physiological conditions in vivo.

It should be noted that there is a close correlation between LepR^+^ cells and hematopoiesis. LepR^+^ cells express HSC niche factors like stem cell factor (SCF) and CXCL12 in a high level, and the ablation of LepR^+^ cells impairs hematopoiesis. Further research unveiled that LepR^+^ cells contribute to hematopoietic regeneration through adipogenic differentiation. The bone marrow adipocytes can synthesize SCF and adiponectin to support hematopoietic stem cell proliferation [78, 79], which is contradictory with the previous view that bone marrow adipocytes act negatively on hematopoiesis [73, 80].

Further experiment revealed that LepR also plays a critical role in regulating the differentiation of SSCs through the Jak2/Stat3 signaling pathway. A high-fat diet or adiposity can activate Lep/LepR signaling, which promotes adipogenesis at the expense of osteogenesis and acts as a negative factor in bone fracture regeneration [81].

### 3.3. Hox11-Expressing Cells

*Hox* genes are comprised of 13 sets of transcription factors that play a critical part in regulating the formation and regeneration of vertebral and limb skeleton and, additionally, differentiation of stem cells [82–84]. *Hox* genes are expressed in a spatiotemporal sequence, which means *Hox1* and *Hox2* appear early and anteriorly while *Hox13* is expressed late and posteriorly. Among them, Hox11 expressed in zeugopod (tibia/fibula and radius/ulna) [85, 86].

Hox11^+^ cells in adult mice are nonendothelial, nonhematopoietic, and undifferentiated cells [86]. They are restricted within zeugopod, specifically speaking, in the periosteal and perivascular areas throughout the adulthood [87]. Most adult Hox11^+^ cells were found to express other classic SSC markers including PDGFR*α*, CD51, and LepR. In addition, perivascular Hox11^+^ cells in adult mice were supposed to represent a group of SSCs due to the following reasons. Firstly, they were clonogenic in vitro and cells positive for Hox11, PDGFR*α*, and CD51 exhibit almost three times greater self-renewal ability than cells only positive for PDGFR*α* and CD51. Secondly, perivascular Hox11^+^ cells were tripotent to give rise to osteoblasts, chondrocytes, and adipocytes in vitro and vivo. Thirdly, Hox11^+^ cells were crucial for fracture repair of zeugopod [88]. They can respond to injury through self-expansion, and they could differentiate into osteoblasts and chondrocytes upon transplantation into fracture callus. Dysfunction of *Hox11* would cause defective fracture repairment, which was reflected in reduced cartilage formation, delayed ossification, and increased adipogenic differentiation of Hox11^+^ cells. Of note, these effects were zeugopod-specific. In other words, fracture healing of other regions was not influenced by function loss of Hox11. Collectively, it is believed that Hox11 can regarded as a marker of SSCs.

## 4. Periosteum

Periosteum is the membrane that lines the outer surface of bones. It can be divided into two layers, and the inner layer is known as a reservoir of osteogenic progenitors, which play an important part in bone formation and bone generation [89]. Considering its easy access and minimal invasiveness, periosteum is supposed to be a good place to find SSCs for clinical treatment [90].

Over the past few years, several markers have been reported for potential identification of SSCs in the periosteum, but due to a low purity and stemness, these markers cannot be used alone [90, 91]. A recent study demonstrated that there exists a pool of SSCs within the periosteum. These cells can give rise to osteocytes, adipocytes, and chondrocytes in vitro. Compared with bone marrow SSCs, the SSCs in the periosteum were more clonogenic and possessed greater ability of cell growth and bone regeneration. More importantly, this pool of SSCs can survive after periosteum grafting. Periostin, a secreted extracellular matrix protein, was believed to be essential for maintaining the pool of periosteal skeletal stem cells [92]. However, a long-standing question impeding translational research is a lack of specific markers for this pool. Until recently, cathepsin K was identified.

Cathepsin K (CTSK) is a lysosomal cysteine protease that mainly secreted by activated osteoclasts [93]. Cathepsin K can play a major part in bone remodeling and resorption by degrading collagen and matrix proteins. Bone resorption can be reversed by inactivation or deletion of *Ctsk*. Thus, *Ctsk* is a recognized marker for marrow mature osteoclasts both in vivo and in vitro [93–95]. In 2013, Yang et al. accidentally identified a pool of *Ctsk^+^* cells within Ranvier's groove. Conditional knockout of tyrosine phosphatase SHP2 in *Ctsk^+^* cells leads to metachondromatosis, a disease characterized by the presence of multiple enchondromas and osteochondromas, indicating that *Ctsk^+^* cells in Ranvier's groove exhibit functional properties consistent with mesenchymal progenitors. They termed these cells as Ctsk^+^ chondroid progenitors (CCPs) [96].

Recently, Debnath et al. discovered that Ctsk could label a type of skeletal stem cells that exist in the periosteal mesenchyme of the long bones or calvarium, termed as periosteal stem cells (PSCs) [97]. Three groups of nonhematopoietic CTSK-mGFP mesenchymal cells were identified: PSCs and periosteal progenitors 1 and 2 (PP1 and PP2), among which only PSCs were constantly positive for CD200 [98]. PSCs can give rise to all the CTSK-mGFP cells, but other cells cannot, namely, PSCs lie at the top of the CTSK-mGFP differentiation hierarchy. Transcriptional analysis and single-cell RNA sequencing showed that PSCs express MSC-related gene. Besides, PSCs possess the ability of self-renewal and multipotency to differentiate into osteoblasts, adipocytes, and chondrocytes. Critically, PSCs can retain these abilities even after serial transplantation into the mammary fat pad and kidney capsule.

PSC-derived osteoblasts were so crucial that lack of it can cause reduced periosteal bone formation and abnormal cortical structure. PSCs can contribute to fracture healing via self-expansion and increased osteogenic and chondrogenic differentiation, which is intriguing as the periosteum is involved in intramembranous instead of endochondral bone formation. Moreover, PSCs isolated from the fracture callus promoted endochondral ossification after ectopic transplantation into the kidney capsule. The plasticity of PSCs partially explains the contradiction.

It is noteworthy that researchers managed to isolate human periosteal stem cells (h-PSCs) in human periosteal tissue of the femur. The h-PSCs were analogous to m-PSCs in immunophenotype and are multipotent both in vivo and in vitro, which provides a feasible way for treating human skeletal disorders.

## 5. Cranial Suture

Different from long bones, craniofacial bones are developed via intramembranous bone formation without intermediate cartilage, indicating that SSCs residing here prefer bone formation to chondrogenesis [99, 100]. Besides, there is little bone marrow space inside of the craniofacial bones compared with the long bones [101]. The gap between craniofacial bones is known as a suture. Premature closure of the suture characterizes craniosynostosis, a developmental craniofacial deformity accompanying with a series of severe consequences including increased intracranial pressure and craniofacial dysmorphism [102]. A cranial suture acts as the growth site for osteogenesis of craniofacial bones, and therefore, suture mesenchyme is postulated as a main source of craniofacial SSCs. The two SSCs we described in the following passage both reside within suture mesenchyme [103].

### 5.1. Gli1-Expressing Cells

Besides as a marker of MMPs (previously described in this review), Gli1 was initially regarded as a marker of MSCs in the cranial suture of adult mice [52]. Cranial Gli1^+^ cells share a lot of characteristics with MMPs. Cranial Gli1^+^ cells are capable of trilineage differentiation (osteogenic, chondrogenic, and adipogenic), but adipogenic differentiation ability of them was not comparable to that of the MMPs. Gli1^+^ cells can contribute to bone injury healing. Besides, they are regulated by Ihh signaling pathways, blockade of which could cause reduced bone volume.

However, compared with MMPs, Gli1^+^ cells in cranial sutures can generate periosteum and dura but contribute little to bone marrow and vasculature. More importantly, Gli1^+^ cells in the suture mesenchyme are crucial for local homeostasis, and ablation of them using diphtheria toxin (DTA) resulted in a typical symptom of craniosynostosis, growth arrest, osteoporosis, and compromised injury repair.

### 5.2. Axin2-Expressing Cells

Axin2, also known as conductin or Axil, is a negative regulator of Wnt/*β*-catenin pathways and thus plays a critical role in skeletogenesis. Axin2 can inhibit intramembranous bone formation, and the inactivation of Axin2 leads to craniosynostosis because of excessive intramembranous ossification [104, 105]. Of note, fate commitment of Axin2 stem cells is tightly regulated by interaction of several signaling pathways including FGF, BMP, and Wnt [106, 107].

Maruyama et al. identified that Axin2 can mark a group of SSCs or specifically termed as suture stem cells (SuSCs) [108]. Axin2^+^ SuSCs and their descendants were restricted within calvarial sutures and nearly absent in long bones, indicating that Axin2^+^ cells represent a totally distinct group of SSCs from those populations in the long bones. Axin2^+^ cells possess the capacity of self-renewal and colony forming and were able to give rise to osteogenic lineages during the developmental period and adulthood of mice. Axin2^+^ cells could strongly respond to bone injury through self-expansion and producing skeletogenic cell types including osteoprogenitors and osteocytes in vivo. Although Axin2^+^ cells did not differentiate into chondrogenic cells under normal conditions, they are committed to cartilage formation with BMP2 induction. Importantly, Axin2^+^ cells showed a great ability of bone regeneration upon implantation into the kidney capsule and they could contribute to the formation of the ectopic bone that appears to share morphological features with calvarial skeletons, which had little marrow structure. Further experiment indicated that the Axin2^+^ cells applied into the injury site can directly engraft into the regenerated bone and promoted osteogenesis.

Axin2^+^ cells in the suture mesenchyme express little markers of MSCs except LepR but overlap a lot with Gli1^+^ cell population. Distinct from Gli1^+^ cells which appear to be located within the whole suture and contribute to calvarial maintenance of the vicinity of central bone plates, Axin2^+^ SuSCs were mainly located in the midline of the suture mesenchyme. The aforementioned differences indicate that the two groups of stem cells contribute to different parts of calvarium.

## 6. Conclusion

In this review, we emphasize four places of bones (growth plate, perivascular areas, periosteum, and cranial suture) as a possible source of SSCs and evaluate these cells from a SSC perspective. Compared with traditional mesenchymal stem cells, these identified “skeletal stem cells” are more defined and therefore more efficient in clinical utility. However, not all of them can differentiate into osteoblasts, chondrocytes, and adipocytes both in vitro and in vivo. More importantly, some markers are not precise enough to represent a pure group of SSCs, resulting from a contamination by their descendants or other cells. Hopefully, this flaw would be alleviated when used in combination with other markers. Looking back on these cells, we notice that different SSCs may share the same markers in space and time. Meanwhile, SSCs exhibit site-specific characteristics, indicating that distinct but somewhat overlapped pools of SSCs contribute to skeletogenesis altogether. In conclusion, to make the best use of SSCs, the mechanism of their fate commitment requires further research.

## Figures and Tables

**Table 1:** Comparison of MSCs and SSCs.

	MSCs	SSCs
Location	Ubiquitously	Skeleton
Skeletal lineage restricted	No	Yes
Homogeneity	Low	High
Stability	Low	High
Multilineage differentiation in vivo	Unpredictable	Yes
Ectopic bone formation	Unpredictable	Yes

## Abbreviations

SSCs: Skeletal stem cells
MSCs: Mesenchymal stromal/stem cells
Mx1: Myxovirus resistance-1
FACS: Fluorescence-activated cell sorting
Ihh: Indian hedgehog
PTHrP: Parathyroid hormone-related protein
FGF: Fibroblast growth factors
BMP: Bone morphogenetic proteins
VEGF: Vascular endothelial growth factor
HSCs: Hematopoietic stem cells
CFU-Fs: Colony-forming unit-fibroblasts
mSSC: Mouse skeletal stem cell
PCPs: Prochondrogenic progenitors
BCSPs: Bone, cartilage, and stromal progenitors
PDPN: Podoplanin
HAS: Human adipose stroma
hOPs: Human osteoprogenitors
hHSCs: Human hematopoietic stem cells
Hh: Hedgehog
Gli1: Glioma-associated oncogene 1
MMPs: Metaphyseal mesenchymal progenitors
BMSCs: Bone marrow stromal cells
P1NP: Propeptide of type I procollagen
CTX-I: C-telopeptide
Grem1: Gremlin1
OCR: Osteochondroreticular
Angpt1: Angiopoietin-1
Sca1: Stem cell antigen-1
OPCs: Osteochondrogenic progenitor cells
APCs: Adipogenic progenitor cells
preAd: Preadipocyte
DPP4: Dipeptidyl peptidase-4
LepR: Leptin receptor
LepR+: Leptin receptor-expressing
SCF: Stem cell factor
CTSK: Cathepsin K
CCPs: Ctsk^+^ chondroid progenitors
PSCs: Periosteal stem cells
PP1: Periosteal progenitor 1
PP2: Periosteal progenitor 2
h-PSCs: Human periosteal stem cells
DTA: Diphtheria toxin
SuSCs: Suture stem cells.

## References

[1] Manilay J. O., Zouali M. (2014). Tight relationships between B lymphocytes and the skeletal system. *Trends in Molecular Medicine*.

[2] Riddle R. C., Clemens T. L. (2017). Bone cell bioenergetics and skeletal energy homeostasis. *Physiological Reviews*.

[3] Watson E. C., Adams R. H. (2018). Biology of bone: the vasculature of the skeletal system. *Cold Spring Harbor Perspectives in Medicine*.

[4] Edwards J. R., Mundy G. R. (2011). Advances in osteoclast biology: old findings and new insights from mouse models. *Nature Reviews Rheumatology*.

[5] Harada S., Rodan G. A. (2003). Control of osteoblast function and regulation of bone mass. *Nature*.

[6] Cummings S. R., Melton L. J. (2002). Epidemiology and outcomes of osteoporotic fractures. *Lancet*.

[7] Lee D. M., Weinblatt M. E. (2001). Rheumatoid arthritis. *Lancet*.

[8] Aghebati-Maleki L., Dolati S., Zandi R. (2018). Prospect of mesenchymal stem cells in therapy of osteoporosis: a review. *Journal of Cellular Physiology*.

[9] Liu W., Zhou L., Zhou C. (2016). GDF11 decreases bone mass by stimulating osteoclastogenesis and inhibiting osteoblast differentiation. *Nature Communications*.

[10] Guo Y. C., Wang M. Y., Zhang S. W. (2018). Ubiquitin-specific protease USP34 controls osteogenic differentiation and bone formation by regulating BMP2 signaling. *The EMBO Journal*.

[11] Fan Y., Hanai J. I., le P. T. (2017). Parathyroid hormone directs bone marrow mesenchymal cell fate. *Cell Metabolism*.

[12] Wu Y., Xie L., Wang M. (2018). Mettl3-mediated m^6^A RNA methylation regulates the fate of bone marrow mesenchymal stem cells and osteoporosis. *Nature Communications*.

[13] Zhou C. C., Xiong Q. C., Zhu X. X. (2017). AFF1 and AFF4 differentially regulate the osteogenic differentiation of human MSCs. *Bone Research*.

[14] Sipp D., Robey P. G., Turner L. (2018). Clear up this stem-cell mess. *Nature*.

[15] Bianco P., Robey P. G., Simmons P. J. (2008). Mesenchymal stem cells: revisiting history, concepts, and assays. *Cell Stem Cell*.

[16] Galipeau J., Weiss D. J., Dominici M. (2019). Response to *Nature* commentary “Clear up this stem-cell mess”. *Cytotherapy*.

[17] Bianco P., Robey P. G. (2015). Skeletal stem cells. *Development*.

[18] Bianco P., Cao X., Frenette P. S. (2013). The meaning, the sense and the significance: translating the science of mesenchymal stem cells into medicine. *Nature Medicine*.

[19] Nusspaumer G., Jaiswal S., Barbero A. (2017). Ontogenic identification and analysis of mesenchymal stromal cell populations during mouse limb and long bone development. *Stem Cell Reports*.

[20] Dominici M., le Blanc K., Mueller I. (2006). Minimal criteria for defining multipotent mesenchymal stromal cells. The International Society for Cellular Therapy position statement. *Cytotherapy*.

[21] Park D., Spencer J. A., Koh B. I. (2012). Endogenous bone marrow MSCs are dynamic, fate-restricted participants in bone maintenance and regeneration. *Cell Stem Cell*.

[22] Caplan A. I. (2005). Review: mesenchymal stem cells: cell-based reconstructive therapy in orthopedics. *Tissue Engineering*.

[23] McLeod C. M., Mauck R. L. (2017). On the origin and impact of mesenchymal stem cell heterogeneity: new insights and emerging tools for single cell analysis. *European Cells & Materials*.

[24] Chan C. K. F., Gulati G. S., Sinha R. (2018). Identification of the human skeletal stem cell. *Cell*.

[25] Chan C. K. F., Seo E. Y., Chen J. Y. (2015). Identification and specification of the mouse skeletal stem cell. *Cell*.

[26] Mohamed F. F., Franceschi R. T. (2017). Skeletal stem cells: origins, functions and uncertainties. *Current Molecular Biology Reports*.

[27] Zhu H., Guo Z. K., Jiang X. X. (2010). A protocol for isolation and culture of mesenchymal stem cells from mouse compact bone. *Nature Protocols*.

[28] Bianco P. (2014). “Mesenchymal” stem cells. *Annual Review of Cell and Developmental Biology*.

[29] Gulati G. S., Murphy M. P., Marecic O. (2018). Isolation and functional assessment of mouse skeletal stem cell lineage. *Nature Protocols*.

[30] Tichy E. D., Mourkioti F. (2018). Human skeletal stem cells: the markers provide some clues in the hunt for hidden treasure. *Cell Stem Cell*.

[31] Kronenberg H. M. (2003). Developmental regulation of the growth plate. *Nature*.

[32] van der Eerden B. C. J., Karperien M., Wit J. M. (2003). Systemic and local regulation of the growth plate. *Endocrine Reviews*.

[33] Long F., Ornitz D. M. (2013). Development of the endochondral skeleton. *Cold Spring Harbor Perspectives in Biology*.

[34] Chung U. I., Schipani E., McMahon A. P., Kronenberg H. M. (2001). Indian hedgehog couples chondrogenesis to osteogenesis in endochondral bone development. *The Journal of Clinical Investigation*.

[35] Horton W. A., Degnin C. R. (2009). FGFs in endochondral skeletal development. *Trends in Endocrinology and Metabolism: TEM*.

[36] Abad V., Meyers J. L., Weise M. (2002). The role of the resting zone in growth plate chondrogenesis. *Endocrinology*.

[37] Newton P. T., Li L., Zhou B. (2019). A radical switch in clonality reveals a stem cell niche in the epiphyseal growth plate. *Nature*.

[38] Kim H., Wrann C. D., Jedrychowski M. (2018). Irisin mediates effects on bone and fat via *α*V integrin receptors. *Cell*.

[39] Lai C. F., Cheng S. L. (2005). *α*v*β* integrins play an essential role in BMP-2 induction of osteoblast differentiation. *Journal of Bone and Mineral Research*.

[40] Thi M. M., Suadicani S. O., Schaffler M. B., Weinbaum S., Spray D. C. (2013). Mechanosensory responses of osteocytes to physiological forces occur along processes and not cell body and require *α*V*β*3 integrin. *Proceedings of the National Academy of Sciences of the United States of America*.

[41] Craig W., Kay R., Cutler R. L., Lansdorp P. M. (1993). Expression of Thy-1 on human hematopoietic progenitor cells. *The Journal of Experimental Medicine*.

[42] Picke A. K., Campbell G. M., Schmidt F. N. (2018). Thy-1 deficiency augments bone loss in obesity by affecting bone formation and resorption. *Frontiers in Cell and Developmental Biology*.

[43] Williams A. F., Barclay A. N. (1988). The immunoglobulin superfamily--domains for cell surface recognition. *Annual Review of Immunology*.

[44] Kassem M., Bianco P. (2015). Skeletal stem cells in space and time. *Cell*.

[45] Astarita J. L., Acton S. E., Turley S. J. (2012). Podoplanin: emerging functions in development, the immune system, and cancer. *Frontiers in Immunology*.

[46] Sacchetti B., Funari A., Michienzi S. (2007). Self-renewing osteoprogenitors in bone marrow sinusoids can organize a hematopoietic microenvironment. *Cell*.

[47] Zhang B. (2010). CD73: a novel target for cancer immunotherapy. *Cancer Research*.

[48] Zannettino A. C., Bühring H. J., Niutta S., Watt S. M., Benton M. A., Simmons P. J. (1998). The sialomucin CD164 (MGC-24v) is an adhesive glycoprotein expressed by human hematopoietic progenitors and bone marrow stromal cells that serves as a potent negative regulator of hematopoiesis. *Blood*.

[49] Lanske B., Karaplis A. C., Lee K. (1996). PTH/PTHrP receptor in early development and Indian hedgehog-regulated bone growth. *Science*.

[50] Mizuhashi K., Ono W., Matsushita Y. (2018). Resting zone of the growth plate houses a unique class of skeletal stem cells. *Nature*.

[51] Hojo H., Ohba S., Yano F. (2012). Gli1 protein participates in Hedgehog-mediated specification of osteoblast lineage during endochondral ossification. *The Journal of Biological Chemistry*.

[52] Zhao H., Feng J., Ho T. V., Grimes W., Urata M., Chai Y. (2015). The suture provides a niche for mesenchymal stem cells of craniofacial bones. *Nature Cell Biology*.

[53] Zhao H., Feng J., Seidel K. (2014). Secretion of shh by a neurovascular bundle niche supports mesenchymal stem cell homeostasis in the adult mouse incisor. *Cell Stem Cell*.

[54] Ye L., Lou F., Yu F. (2018). NUMB maintains bone mass by promoting degradation of PTEN and GLI1 via ubiquitination in osteoblasts. *Bone Research*.

[55] Shi Y., He G., Lee W. C., McKenzie J. A., Silva M. J., Long F. (2017). Gli1 identifies osteogenic progenitors for bone formation and fracture repair. *Nature Communications*.

[56] Song L., Liu M., Ono N., Bringhurst F. R., Kronenberg H. M., Guo J. (2012). Loss of wnt/*β*-catenin signaling causes cell fate shift of preosteoblasts from osteoblasts to adipocytes. *Journal of Bone and Mineral Research*.

[57] Khokha M. K., Hsu D., Brunet L. J., Dionne M. S., Harland R. M. (2003). Gremlin is the BMP antagonist required for maintenance of Shh and Fgf signals during limb patterning. *Nature Genetics*.

[58] Canalis E., Parker K., Zanotti S. (2012). Gremlin1 is required for skeletal development and postnatal skeletal homeostasis. *Journal of Cellular Physiology*.

[59] Merino R., Rodriguez-Leon J., Macias D., Gañan Y., Economides A. N., Hurle J. M. (1999). The BMP antagonist Gremlin regulates outgrowth, chondrogenesis and programmed cell death in the developing limb. *Development*.

[60] Worthley D. L., Churchill M., Compton J. T. (2015). Gremlin 1 identifies a skeletal stem cell with bone, cartilage, and reticular stromal potential. *Cell*.

[61] Méndez-Ferrer S., Michurina T. V., Ferraro F. (2010). Mesenchymal and haematopoietic stem cells form a unique bone marrow niche. *Nature*.

[62] Zhou B. O., Yue R., Murphy M. M., Peyer J. G., Morrison S. J. (2014). Leptin-receptor-expressing mesenchymal stromal cells represent the main source of bone formed by adult bone marrow. *Cell Stem Cell*.

[63] Zhao M., Tao F., Venkatraman A. (2019). N-cadherin-expressing bone and marrow stromal progenitor cells maintain reserve hematopoietic stem cells. *Cell Reports*.

[64] Crisan M., Yap S., Casteilla L. (2008). A perivascular origin for mesenchymal stem cells in multiple human organs. *Cell Stem Cell*.

[65] Shi S., Gronthos S. (2003). Perivascular niche of postnatal mesenchymal stem cells in human bone marrow and dental pulp. *Journal of Bone and Mineral Research*.

[66] Chan C. K. F., Chen C.-C., Luppen C. A. (2009). Endochondral ossification is required for haematopoietic stem-cell niche formation. *Nature*.

[67] Greenbaum A., Hsu Y. M. S., Day R. B. (2013). CXCL12 in early mesenchymal progenitors is required for haematopoietic stem-cell maintenance. *Nature*.

[68] Pinho S., Lacombe J., Hanoun M. (2013). PDGFR*α* and CD51 mark human nestin+sphere-forming mesenchymal stem cells capable of hematopoietic progenitor cell expansion. *The Journal of Experimental Medicine*.

[69] Boxall S. A., Jones E. (2012). Markers for characterization of bone marrow multipotential stromal cells. *Stem Cells International*.

[70] Holmes C., Stanford W. L. (2007). Concise review: stem cell antigen-1: expression, function, and enigma. *Stem Cells*.

[71] Tan Y., Zhao M., Xiang B., Chang C., Lu Q. (2016). CD24: from a hematopoietic differentiation antigen to a genetic risk factor for multiple autoimmune diseases. *Clinical Reviews in Allergy and Immunology*.

[72] Ambrosi T. H., Scialdone A., Graja A. (2017). Adipocyte accumulation in the bone marrow during obesity and aging impairs stem cell-based hematopoietic and bone regeneration. *Cell Stem Cell*.

[73] Naveiras O., Nardi V., Wenzel P. L., Hauschka P. V., Fahey F., Daley G. Q. (2009). Bone-marrow adipocytes as negative regulators of the haematopoietic microenvironment. *Nature*.

[74] Flier J. S., Maratos-Flier E. (2017). Leptin’s physiologic role: does the emperor of energy balance have no clothes?. *Cell Metabolism*.

[75] Turner R. T., Kalra S. P., Wong C. P. (2013). Peripheral leptin regulates bone formation. *Journal of Bone and Mineral Research*.

[76] Takeda S., Elefteriou F., Levasseur R. (2002). Leptin regulates bone formation via the sympathetic nervous system. *Cell*.

[77] Clément K., Vaisse C., Lahlou N. (1998). A mutation in the human leptin receptor gene causes obesity and pituitary dysfunction. *Nature*.

[78] DiMascio L., Voermans C., Uqoezwa M. (2007). Identification of adiponectin as a novel hemopoietic stem cell growth factor. *Journal of Immunology*.

[79] Zhou B. O., Yu H., Yue R. (2017). Bone marrow adipocytes promote the regeneration of stem cells and haematopoiesis by secreting SCF. *Nature Cell Biology*.

[80] Li Q., Wu Y., Kang N. (2018). Marrow adipose tissue: its origin, function, and regulation in bone remodeling and regeneration. *Stem Cells International*.

[81] Yue R., Zhou B. O., Shimada I. S., Zhao Z., Morrison S. J. (2016). Leptin receptor promotes adipogenesis and reduces osteogenesis by regulating mesenchymal stromal cells in adult bone marrow. *Cell Stem Cell*.

[82] Gonzalez-Martin M. C., Mallo M., Ros M. A. (2014). Long bone development requires a threshold of Hox function. *Developmental Biology*.

[83] Seifert A., Werheid D. F., Knapp S. M., Tobiasch E. (2015). Role of Hox genes in stem cell differentiation. *World Journal of Stem Cells*.

[84] Leucht P., Kim J. B., Amasha R., James A. W., Girod S., Helms J. A. (2008). Embryonic origin and Hox status determine progenitor cell fate during adult bone regeneration. *Development*.

[85] Wellik D. M., Capecchi M. R. (2003). *Hox10* and *Hox11* genes are required to globally pattern the mammalian skeleton. *Science*.

[86] Swinehart I. T., Schlientz A. J., Quintanilla C. A., Mortlock D. P., Wellik D. M. (2013). *Hox11* genes are required for regional patterning and integration of muscle tendon and bone. *Development*.

[87] Rux D. R., Song J. Y., Swinehart I. T. (2016). Regionally restricted Hox function in adult bone marrow multipotent mesenchymal stem/stromal cells. *Developmental Cell*.

[88] Rux D. R., Song J. Y., Pineault K. M. (2017). *Hox11* function is required for region-specific fracture repair. *Journal of Bone and Mineral Research*.

[89] Allen M. R., Hock J. M., Burr D. B. (2004). Periosteum: biology, regulation, and response to osteoporosis therapies. *Bone*.

[90] Kim Y. K., Nakata H., Yamamoto M., Miyasaka M., Kasugai S., Kuroda S. (2016). Osteogenic potential of mouse periosteum-derived cells sorted for CD90 in vitro and in vivo. *Stem Cells Translational Medicine*.

[91] Matthews B. G., Grcevic D., Wang L. (2014). Analysis of *α*SMA-labeled progenitor cell commitment identifies notch signaling as an important pathway in fracture healing. *Journal of Bone and Mineral Research*.

[92] Duchamp de Lageneste O., Julien A., Abou-Khalil R. (2018). Periosteum contains skeletal stem cells with high bone regenerative potential controlled by Periostin. *Nature Communications*.

[93] Lotinun S., Kiviranta R., Matsubara T. (2013). Osteoclast-specific cathepsin K deletion stimulates S1P-dependent bone formation. *The Journal of Clinical Investigation*.

[94] Panwar P., Xue L., Søe K. (2017). An ectosteric inhibitor of cathepsin K inhibits bone resorption in ovariectomized mice. *Journal of Bone and Mineral Research*.

[95] Bonnet N., Brun J., Rousseau J. C., Duong L. T., Ferrari S. L. (2017). Cathepsin K controls cortical bone formation by degrading periostin. *Journal of Bone and Mineral Research*.

[96] Yang W., Wang J., Moore D. C. (2013). Ptpn11 deletion in a novel progenitor causes metachondromatosis by inducing hedgehog signalling. *Nature*.

[97] Debnath S., Yallowitz A. R., McCormick J. (2018). Discovery of a periosteal stem cell mediating intramembranous bone formation. *Nature*.

[98] Chan C. K. F., Lindau P., Jiang W. (2013). Clonal precursor of bone, cartilage, and hematopoietic niche stromal cells. *Proceedings of the National Academy of Sciences of the United States of America*.

[99] Mao J. J., Prockop D. J. (2012). Stem cells in the face: tooth regeneration and beyond. *Cell Stem Cell*.

[100] Chai Y., Maxson R. E. (2006). Recent advances in craniofacial morphogenesis. *Developmental Dynamics*.

[101] Yang M., Zhang H., Gangolli R. (2014). Advances of mesenchymal stem cells derived from bone marrow and dental tissue in craniofacial tissue engineering. *Current Stem Cell Research & Therapy*.

[102] Morriss-Kay G. M., Wilkie A. O. M. (2005). Growth of the normal skull vault and its alteration in craniosynostosis: insights from human genetics and experimental studies. *Journal of Anatomy*.

[103] Opperman L. A. (2000). Cranial sutures as intramembranous bone growth sites. *Developmental Dynamics*.

[104] Yu H. M., Jerchow B., Sheu T. J. (2005). The role of Axin2 in calvarial morphogenesis and craniosynostosis. *Development*.

[105] Liu B., Yu H. M. I., Hsu W. (2007). Craniosynostosis caused by Axin2 deficiency is mediated through distinct functions of *β*-catenin in proliferation and differentiation. *Developmental Biology*.

[106] Maruyama T., Mirando A. J., Deng C. X., Hsu W. (2010). The balance of WNT and FGF signaling influences mesenchymal stem cell fate during skeletal development. *Science Signaling*.

[107] Maruyama T., Jiang M., Abbott A. (2017). Rap1b is an effector of Axin2 regulating crosstalk of signaling pathways during skeletal development. *Journal of Bone and Mineral Research*.

[108] Maruyama T., Jeong J., Sheu T. J., Hsu W. (2016). Stem cells of the suture mesenchyme in craniofacial bone development, repair and regeneration. *Nature Communications*.

